# Tobacco Alkaloid Assessment in a DSS-Induced Colitis Mouse Model with a Fully Humanized Immune System

**DOI:** 10.3390/ijms24076419

**Published:** 2023-03-29

**Authors:** Catherine Verhaeghe, Marja Talikka, Alain Sewer, Nicolas Sierro, Mehdi Auberson, Dariusz Peric, David Bornand, Remi Dulize, Emmanuel Guedj, Patrick Nef, Sebastien P. Tabruyn, Julia Hoeng, Manuel C. Peitsch, Giuseppe Lo Sasso

**Affiliations:** 1 TransCure bioServices, Av Marie Curie 260, FR-74160 Archamps, France; catherine.verhaeghe@tcbioservices.com (C.V.);; 2 PMI R&D, Philip Morris Products S.A., Quai Jeanrenaud 5, CH-2000 Neuchâtel, Switzerland; marja.talikka@pmi.com (M.T.);

**Keywords:** ulcerative colitis, alkaloids, mouse model, nicotine

## Abstract

Inflammatory bowel disease (IBD) refers to chronic intestinal immune-mediated diseases including two main disease manifestations: ulcerative colitis (UC) and Crohn’s disease (CD). Epidemiological, clinical, and preclinical evidence has highlighted the potential anti-inflammatory properties of naturally occurring alkaloids. In the present study, we investigated the potential anti-inflammatory activities of the tobacco alkaloids nicotine and anatabine in a dextran sulfate sodium (DSS)-induced UC mouse model with a fully humanized immune system. Our results show that nicotine significantly reduced all acute colitis symptoms and improved colitis-specific endpoints, including histopathologically assessed colon inflammation, tissue damage, and mononuclear cell infiltration. The tobacco alkaloid anatabine showed similar effectiveness trends, although they were generally weaker or not significant. Gene expression analysis in the context of biological network models of IBD further pinpointed a possible mechanism by which nicotine attenuated DSS-induced colitis in humanized mice. The current study enables further investigation of possible molecular mechanisms by which tobacco alkaloids attenuate UC symptoms.

## 1. Introduction

Inflammatory bowel disease (IBD) is the collective term for chronic idiopathic immune-mediated diseases characterized by chronic and relapsing inflammation of the gastrointestinal tract. The incidence and prevalence of IBD have increased worldwide with a stable rise expected over the next decade, which will substantially increase the burden on health care systems and society [1,2]. IBD comprises two main disease manifestations, ulcerative colitis (UC) and Crohn’s disease (CD), each having distinctive clinical and pathological features [3,4]. Disease pathogenesis is multifactorial, involving environmental triggers (e.g., drug use, stress, diet, and smoking), genetic predisposition, epithelial barrier defects, and dysregulated interaction between the host immune system and gut microbiome.

The correlation between cigarette smoking and IBD has been established by several epidemiological studies [5]. While smoking is associated with greater disease activity in CD patients [6], there seems to be an inverse association between active smoking and the risk, development, progression, relapse rate, and course of UC [7]. While the effects of smoking on UC are not completely understood, modulation of the mucosal immune response, alterations in intestinal cytokine and eicosanoid levels, and modification of gut permeability have been linked to the nicotine-dependent activation of the “nicotinic anti-inflammatory pathway” via α7-nicotinic acetylcholine receptors (nAChRα7) [7,8,9,10,11,12]. Nicotine also belongs to the pyridine-like class of alkaloids, which are a class of amino acid nitrogen-containing compounds present in different living organisms [13] that have been recently highlighted as candidate drugs to treat intestinal inflammation [14,15].

Several preclinical and clinical studies have been published in which nicotine was administered to mice or patients with UC in different formulations using various administration routes [12,16,17,18,19,20]. The majority of the clinical studies concluded that transdermal nicotine could induce remission in patients with UC compared with placebo patches [12,17]. These observations were recently summarized in a review evaluating the efficacy of transdermal nicotine as a treatment option for mild to moderately active UC. Contrasting results were observed when nicotine was administered by enema [21]. Similar to clinical studies, mouse studies underscored the importance of the administration route. Indeed, only oral nicotine treatment was shown to be effective in dextran sulfate sodium (DSS)-induced colitis; neither subcutaneous injection nor instillation via minipumps affected colitis in the same model [22,23,24]. Furthermore, increasing evidence suggests that other tobacco alkaloids may carry anti-inflammatory properties potentially suitable for the treatment of intestinal inflammation [15,25,26,27]. For instance, studies have suggested anatabine—a minor alkaloid with a chemical structure closely resembling that of nicotine and found in tobacco [28]—could constitute a potential candidate compound for anti-inflammatory drug development. The biological activity of anatabine has been poorly characterized compared with nicotine, but recent publications have shown anatabine-dependent inhibition of pro-inflammatory gene expression induced by lipopolysaccharide in SY-SY5Y, HEK293, human microglia, and human blood mononuclear cells, as well as in the brain and spleen of mouse models of autoimmune encephalomyelitis and Alzheimer’s disease [29,30]. Finally, we have demonstrated potential anatabine-dependent, anti-inflammatory properties in both in vitro and in vivo models of intestinal inflammation [15,31,32,33].

In the present study, to increase the human translatability of a colitis mouse model and investigate the potential role of tobacco alkaloids in reducing intestinal inflammation, we combined a mouse model with a fully humanized immune system and a validated DSS-induced mouse model of UC with disease-specific endpoints and system-wide molecular profiling.

## 2. Results

### 2.1. Full Reconstitution of the Human Immune System in Mouse

**Figure 1:**
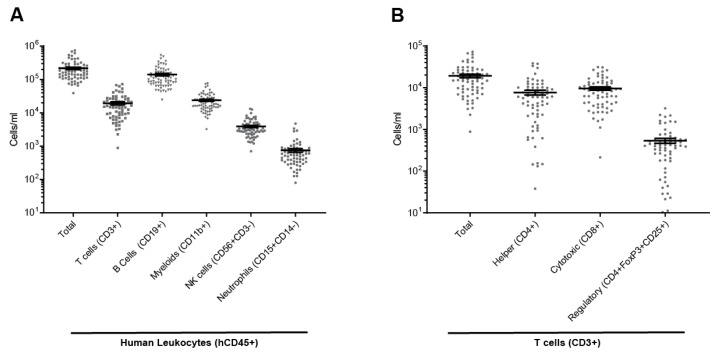
Human immune cell population in the peripheral blood of hu-mice. (**A**) Total human leukocytes (hCD45+), T cells (hCD3+), B cells (hCD19+), myeloid (hCD11b+), NK cells (hCD3-hCD56+), and neutrophils (CD145+CD14−) in the peripheral blood. (**B**) T cells including helper (CD4+), cytotoxic (CD8+), and regulatory (CD4+FoxP3+CD25+) T cells in peripheral blood. Individual values expressed as number of cells per ml, mean ± SEM.

To mimic the contributions of the human immune system in a preclinical model, we engrafted immunodeficient mice (NCG) with cord-blood-derived human CD34+ hematopoietic stem cells and progenitor cells. Fourteen weeks after stem cell injection, engraftment level was monitored with flow cytometry analysis of human CD45+ cells among total blood leukocytes (mouse and human). Only mice with a humanization rate > 30% were used in the subsequent experiments (hu-mice). The average humanization rate was 52.9%. The engrafted human stem cells matured and developed a fully functional human immune system containing mostly B and T lymphocytes, but also monocytes, natural killer (NK) cells, and neutrophils (Figure 1A); T lymphocytes comprised helper, cytotoxic, and regulatory T cells (Figure 1B).

### 2.2. Nicotine and Anatabine Reduce DSS-Induced UC Symptoms

The mice were treated with nicotine or anatabine for 2 weeks before colitis induction by DSS until the end of the experimental phases at day 7 (acute phase) or 12 (7 days DSS + 5 days replacing DSS with water ad libitum—recovery phase).

Treatment with alkaloids did not induce significant body weight loss before DSS induction. Overall survival was recorded during the 12-day experiment. Mice treated with nicotine did not show DSS-induced mortality, but five mice in the vehicle (drinking water) and three mice in the anatabine arms died by the end of the experimental recovery phase (day 12) (Supplementary Figure S1).

**Figure 2:**
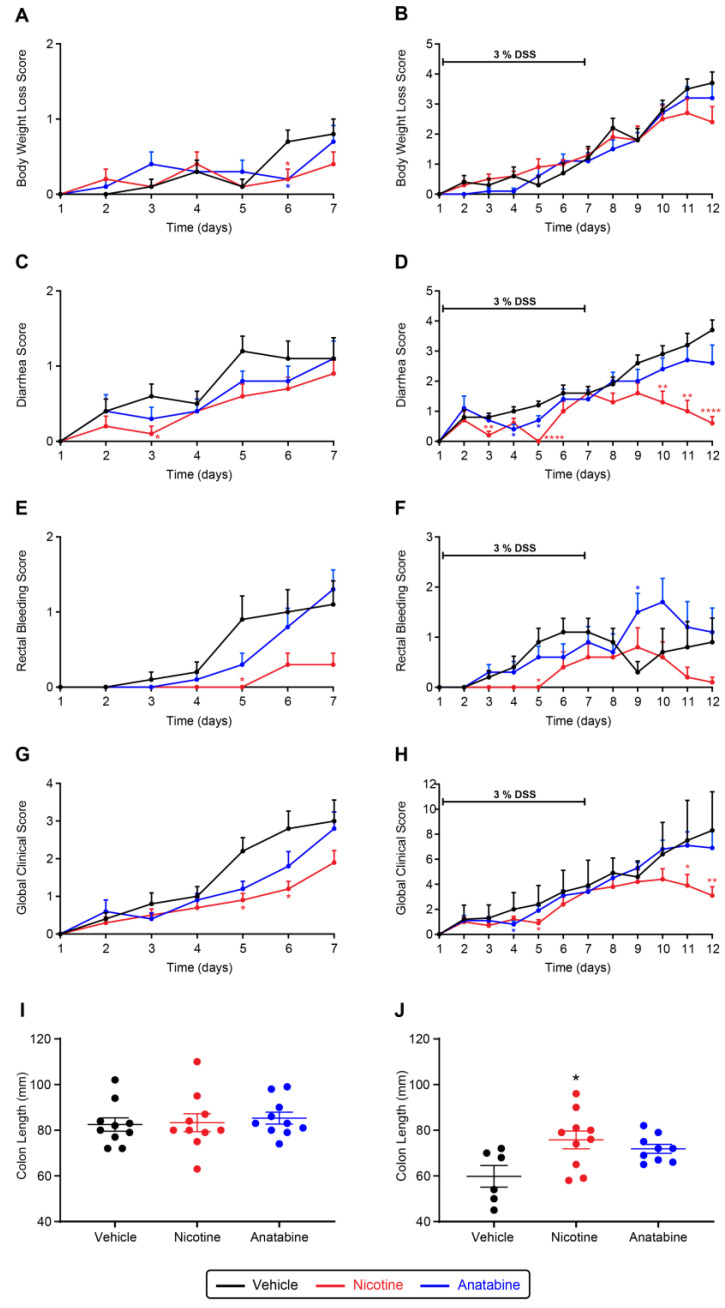
Effect of tobacco alkaloids on clinical parameters of DSS-induced colitis. (**A**,**C**,**E**,**G**,**I**) acute phase; (**B**,**D**,**F**,**H**,**J**) recovery phase. Body weight loss (**A**,**B**), diarrhea (**C**,**D**), rectal bleeding (**E**,**F**), and global clinical score; (**G**,**H**) evolution of DSS-induced hu-mice. Individual colon lengths (mm) with average and SEM measured after sacrifice (**I**,**J**). Results are expressed as mean ± SEM. * *p* ≤ 0.05, ** *p* ≤ 0.01, **** *p* ≤ 0.0001 compared to vehicle (* red: nicotine vs. vehicle, * blue: anatabine vs. vehicle). Acute phase (**A**,**C**,**E**,**G**,**I**): n = 10 for all groups. Recovery phase (**B**,**D**,**F**,**H**,**J**) n = 6 or 9 for vehicle and anatabine, respectively; n = 10 for nicotine.

After colitis induction, body weight started to decrease and reached a loss of 22% in the vehicle-treated group. Anatabine and nicotine attenuated body weight loss, albeit not significantly (Figure 2A,B and Supplementary Figure S2A,B). Diarrhea appeared on day 2 and reached a maximum severity by day 12 (Figure 2C,D). Nicotine treatment significantly reduced diarrhea symptoms during the colitis initiation phase (days 3 and 5) and during the washout period (days 10, 11, and 12) (Figure 2C,D). Anatabine had significant effects only during the initiation phase (days 4 and 5) of the colitis. The areas under curve (AUCs) of the diarrhea score (Supplementary Figure S2C,D) confirmed that nicotine significantly reduced diarrhea in comparison to the vehicle and confirmed that anatabine had the same tendency, although the effect was milder and not significant. In the vehicle-treated group, rectal bleeding appeared on day 3 and peaked by day 6. Rectal bleeding was delayed until day 6 in the nicotine-treated group, and the score remained lower than that of the vehicle-treated group until sacrifice at day 12 (Figure 2E,F). The rectal bleeding AUC confirmed that nicotine significantly reduced bleeding, especially during the initiation phase, with a less pronounced reduction during the washout period (Supplementary Figure S2E,F). Anatabine also showed a trend toward reduced rectal bleeding during the initiation phase, but it appeared to exacerbate symptoms during the washout period. Body weight loss, diarrhea, and rectal bleeding scoring were then combined to calculate a global clinical colitis score. Nicotine significantly reduced the global colitis score induced by DSS in humanized mouse model, and a similar but weaker effect was observed in response to anatabine treatment (Figure 2G,H and Supplementary Figure S2G,H). Finally, following sacrifice at day 7 or 12, colons were dissected and measured before hematoxylin and eosin (HE) staining. Colon shortening is a hallmark of inflammation occurring in the organ. Colon lengths were not different between groups by day 7 (Figure 2I); however, by day 12, mean colon length in the vehicle-treated group (mean of 59.8 mm) was significantly shorter than in the nicotine-treated group (mean of 75.8 mm). Differences in colon lengths between the vehicle- and anatabine-treated (mean of 71.9 mm) groups were not significant (Figure 2J).

### 2.3. Nicotine Reduces Tissue Damage and Inflammation

**Figure 3:**
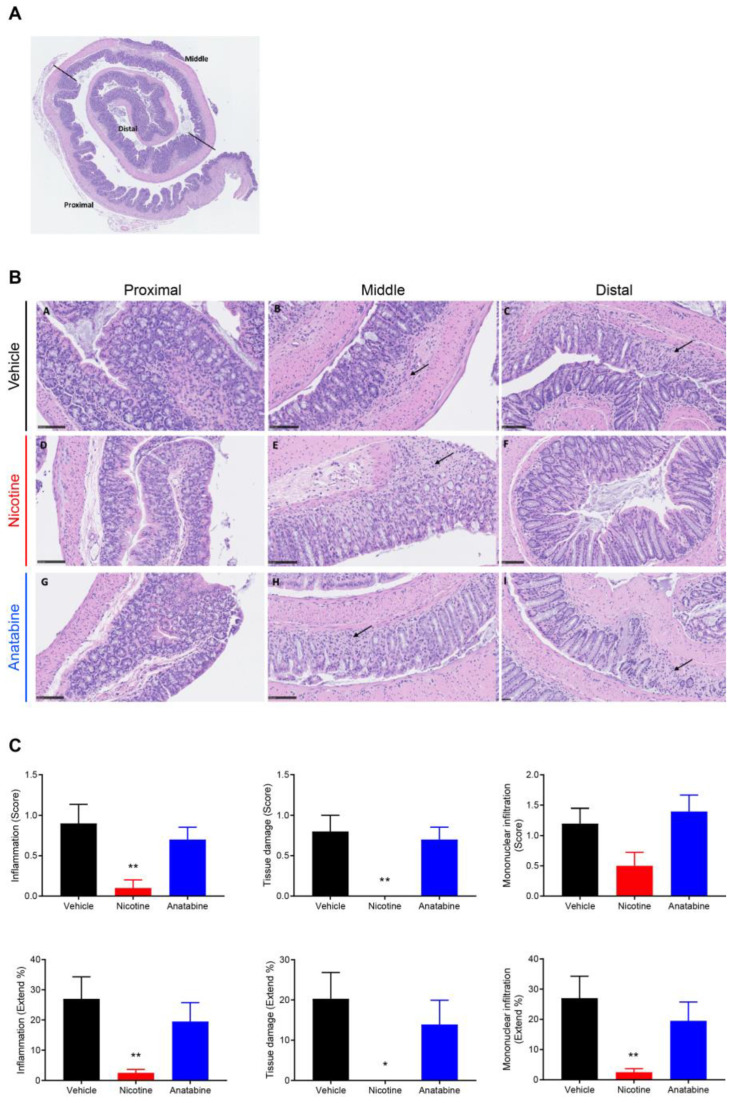
Nicotine reduces histological features of UC at D7. (**A**) Representative HE Swiss-roll colon staining showing the selected proximal, middle, and distal parts. (**B**) Representative HE colon staining showing representative inflammatory foci (arrow) for the three parts of the colon (B-A, B-D, B-G = proximal, B-B, B-E, B-H = middle, and B-C, B-F, B-I = distal) in the three groups (B-A, B-B, B-C = vehicle, B-D, B-E, B-F = nicotine, and B-G, B-H, B-I = anatabine). Scale bar = 100 µm. (**C**) Inflammation (left panels), tissue damage (middle panels), and mononuclear cell infiltration (right panel) score (upper panels) and extent (lower panels) in the distal colon. Mice were sacrificed at the end of DSS administration (D7) for histology assessment. Mean ± SEM are shown. * *p* ≤ 0.05, ** *p* ≤ 0.01. n = 10 for all groups.

The three regions (proximal, middle, and distal) in the Swiss-roll colon samples (Figure 3A) were qualitatively assessed for inflammation, tissue damage, and mononuclear cell infiltration. These three variables were scored, and a percentage of surface was calculated (Figure 3B,C). At day 7, inflammation, tissue damage, and mononuclear cell infiltration scores and extents were significantly reduced in the distal part of the colon in the nicotine-treated mice compared with the vehicle group (Figure 3C). At day 12, the reduced histological scores in the nicotine compared with the vehicle group were not statistically significant (Supplementary Figure S3). Anatabine did not reduce these three parameters at day 7 or day 12.

### 2.4. Effects of Nicotine and Anatabine on Circulating and Intestinal Human Immune Cells

**Figure 4:**
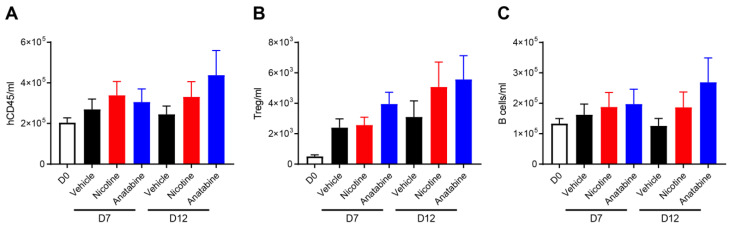
Anatabine increases B-cell content in peripheral blood. Absolute counts of (A) total human immune cells (hCD45), (B) Treg (hCD45+hCD3+hFoxP3+hCD25+), and (C) B cells (hCD45+CD19+) in peripheral blood before UC induction (D0) and at sacrifice (D7 and D12). Individual values expressed as number of cells per ml, mean ± SEM. D7: n = 10 for all groups. D12: n = 7 for vehicle and anatabine; n = 10 for nicotine.

Flow cytometry was performed to determine the effects of nicotine and anatabine on human immune cell populations in the blood samples collected at days 7 and 12. The number of circulating human immune cells (hCD45+) was not significantly affected by treatments on day 7 or on day 12 (Figure 4A). The numbers of regulatory T cells (Tregs) increased after colitis induction, and this increase was more pronounced in nicotine- and anatabine-treated animals at day 12, albeit not significantly (Figure 4B). The number of B cells was not significantly altered by colitis induction (Figure 4C, D0 vs. vehicle) or treatments. Finally, no significant difference was observed in the number of monocytes or neutrophils in any of the groups at day 7 or 12 compared with before colitis induction.

**Figure 5:**
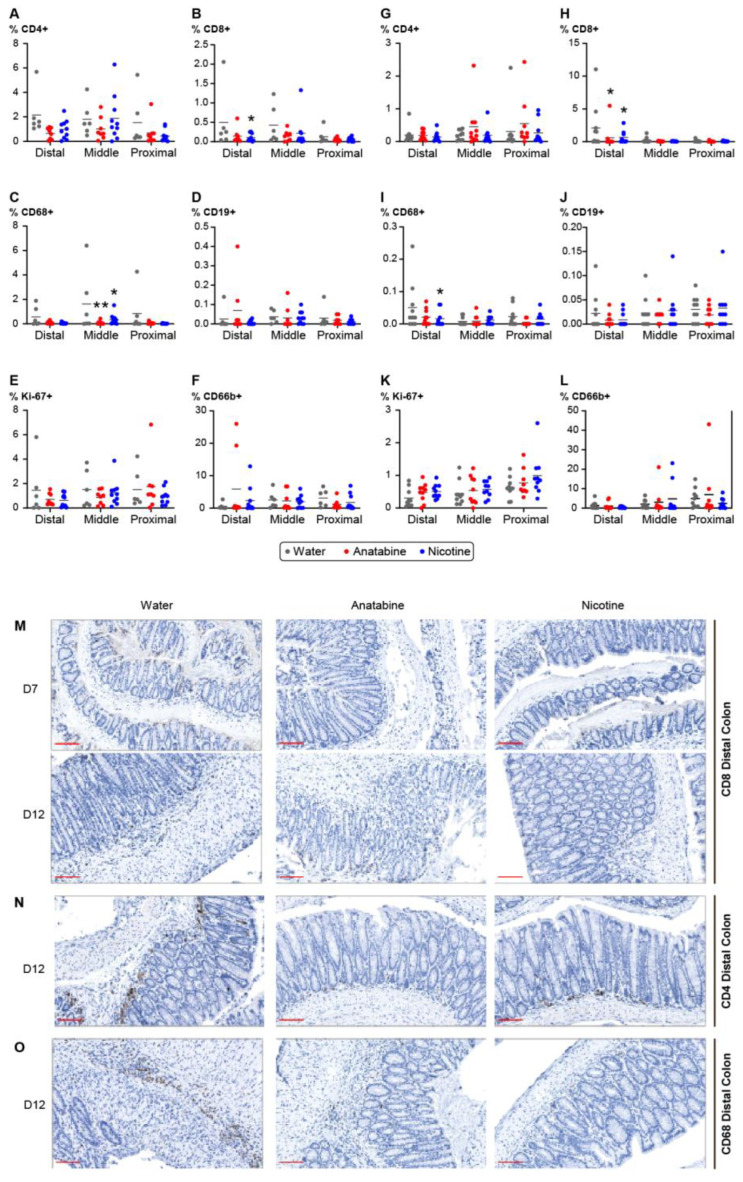
Nicotine- and anatabine-treated mice colon exhibited decreased immune cell infiltration. (**A**–**L**) Percentage of positive cells corresponds to the cells detected by the antibody normalized to the total cell number in the DSS recovery (**A**–**F**) and acute (**G**–**L**) groups. (**A**,**G**) Percentage of CD4+ positive T cells, (**B**,**H**) percentage of CD8+ positive T cells, (**C**,**I**) percentage of macrophages, (**D**,**J**) percentage of B cells, (**E**,**K**) percentage of proliferative cells detected with anti-Ki67 antibody, (**F**,**L**) percentage of neutrophils. Individual values and means are shown. Two-way ANOVA with Sidak’s multiple comparison test was used for analysis. * *p* < 0.05, ** *p* < 0.01 treatment versus water. (**M**–**O**) Representative pictures of colon staining in the anatabine and nicotine treatment groups. (**M**) Colon staining of CD8+ T cells at D7 and D12. (**N**) Colons staining of CD4+ T cells at D12. (**O**) Colon staining of macrophages with anti-CD68 antibody at D12. Scale bar = 100 mm. D7: n = 10 for all groups. D12: n = 6 or 8 for vehicle and anatabine, respectively; n = 10 for nicotine.

To assess human immune cell infiltration in the colon of DSS-treated hu-mice, the fixed colons were stained to label T cells, B cells, macrophages, neutrophils, and proliferative cells (Figure 5). Analysis of colon infiltration at day 12 showed a reduction in CD8+ T cells in the distal part of the colon of mice treated with nicotine compared with the vehicle (Figure 5B). A significant reduction in macrophages was also found in the middle part of the colon of anatabine- and nicotine-treated mice (Figure 5C). Notably, the proportion of proliferative cells did not change after treatment with nicotine or anatabine (Figure 5E). In the DSS acute groups (Figure 5G–L), only a significant reduction in CD8+ T cells and a reduction in macrophages were found in anatabine- and nicotine-treated mice (Figure 5H,I). Taken together, the reductions in T cells and macrophages at D12 and D7 in the distal and middle parts of the colon of anatabine- and nicotine-treated mice correlated with the reduction in colitis symptoms.

### 2.5. Transcriptomic Data Analysis of Effects of Nicotine and Anatabine on the Distal Colon of DSS-Treated Mice

As the samples used for RNA sequencing contained material from both mouse and human origins, we set up a dedicated preprocessing pipeline for the raw sequencing data (see Section 4.9). Supplementary Figure S4 shows the successful results of this preprocessing step: panel A indicates that the relative abundance between human and mouse RNA was approximately 1 to 500, and panels B and C confirm that the human and mouse RNA originated from the expected tissue types: immune cells for human and intestine for mouse. Consequently, the human-mouse orthologous genes, which represent a sizable fraction of the detected genes (>90%), did not suffer from interspecies contamination and could be safely analyzed downstream to provide data to support the biological interpretation presented in the remainder of this subsection.

To obtain mechanistic insight into nicotine’s effects on DSS-induced colitis, we used an enrichment approach that relies on a database of over 800 molecular entities or nodes (e.g., protein activities, complexes, chemicals) with mRNAs known to be regulated by the nodes. The activity of these nodes can be inferred by comparing differential mRNA regulation in experimental data to the mRNAs assigned under each node, taking into consideration the expected directionality of the regulation [34,35,36]. Hence, the nodes represent inferred changes in protein activity rather than at the mRNA level. Our analysis focused on the nodes that were significantly inferred to be regulated by nicotine at day 6, which showed the clearest effects at the other endpoints. Previously published biological network models were used to connect the regulated nodes and include molecular pathways involved in barrier defense, inflammation, and wound healing in the colon [34]. We also eliminated the network edges that were not causally consistent; i.e., the directionality of inferred regulation of the downstream node was not aligned with its upstream node based on the causal edge directionality between them. All nodes present in the IBD network models are listed with the adjusted *p*-value and directionality of the inferred regulation by nicotine at day 6 in Supplementary File S1.

**Figure 6:**
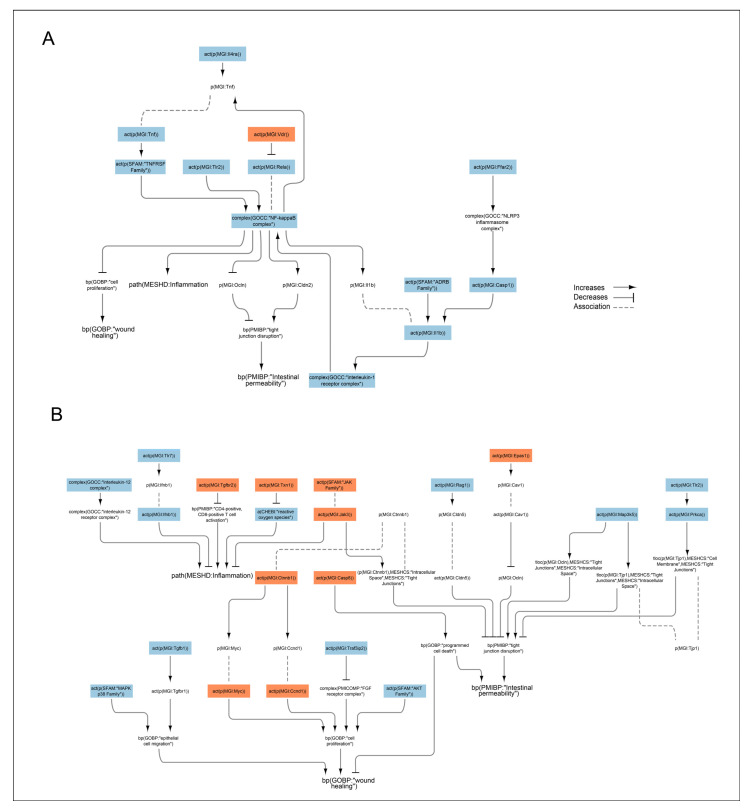
Signaling pathways impacted by nicotine in the DSS-treated humanized mouse colon. The subgraph extracted from the merged IBD network model [34] shows the nodes that were inferred to be regulated with an adjusted *p* < 0.05 when scored with comparative transcriptomic data from nicotine + DSS vs. DSS-treated mouse colon. (**A**) Pathways regulating nuclear factor-κ-light-chain-enhancer of activated B cells (NF-κB) complex. (**B**) Other pathways beside NF-κB inferred to be regulated by nicotine and leading to intestinal permeability, wound healing, and inflammation. The directionalities are shown as orange or blue bars for inferred upregulation or downregulation, respectively. The BEL (biological expression language) terms [37,38] are as follows: act, protein activity; p, protein abundance; bp, biological process; tloc, translocation. MGI is the namespace for mouse proteins, GOCC is the namespace for protein complexes, MESHCS is the namespace for cell structures, MESHD is the namespace for diseases, and SFAM and PMIBP are custom namespaces for protein families and biological processes, respectively. ADRB, adrenoceptor beta; Ctnnb, beta catenin; Casp, caspase; CCnd1, Cyclin D1; Cldn, claudin; Epas, endothelial PAS domain protein; Ffar2, the free fatty acid receptor; Ifn, interferon; Il, interleukin; Jak, Janus kinase; Map3k5, mitogen-activated protein kinase 5; MAPKp38; p38 mitogen-activated protein kinase; NF-kappaB, nuclear factor kappa-light-chain-enhancer of activated B-cell complex; NLRP3, NLR family pyrin domain containing 3; Ocln, occludin, Prkc, protein kinase C; Rag, recombination activating; Tgfb, transforming growth factor beta receptor; Tjp, tight junction protein; Tlr, Toll-like receptor; Tnf, tumor necrosis factor; TNFRSF, TNF receptor superfamily; Traf3ip2, Traf3 interacting protein; Txn, thioredoxin; Vdr, vitamin D receptor.

First, we investigated the pathways regulating nuclear factor-κ-light-chain-enhancer of activated B cells (NF-κB) complex—inferred to be downregulated—in the merged IBD network model (Figure 6A). Along with Toll-like receptor (Tlr) 2, other pathways leading to the inferred downregulation of NF-κB included the inferred upregulation of the vitamin D receptor (Vdr), which negatively regulates Rela, a component of the NF-κB complex. The inferred downregulation of the NF-κB complex was reflected in decreased tumor necrosis factor (Tnf) and interleukin (Il)1b signaling. Tnf production was also be reduced by the inferred downregulation of the Il4 receptor a (ra), and Il1b received negative inputs from the free fatty acid receptor 2 (Ffar2), caspase (Casp)1, and the adrenoceptor beta (ADRB) family. The inferred downregulation of the NF-κB complex suggested decreased tight junction disruption and inflammation and increased wound healing (Figure 6B).

Next, we investigated the impact of other pathways inferred to be regulated by nicotine in the DSS background and leading to intestinal permeability, wound healing, and inflammation. The inferred upregulation of Janus kinase (Jak) and downregulation of mitogen-activated protein kinase (Map3k)5 led to reduced intestinal permeability in nicotine-treated mice via increased translocation of beta catenin (Ctnnb)1 from intracellular space to tight junctions and decreased translocation of occludin (Ocln) and the tight junction protein (Tjp)1 from tight junctions to extracellular space, respectively (Figure 6B). Interestingly, Casp8 and the endothelial PAS domain protein (Epas)1 were inferred to be upregulated, leading to increased cell death and decreased Ocln (an important protector of tight junctions), respectively, indicating increased intestinal permeability. Moreover, recombination-activating gene-1 (Rag1) was inferred to be downregulated, leading to decreased claudin 5 (Cldn5) and indicating increased intestinal permeability. In addition to activating the NF-κB complex, Tlr2 signaling positively regulates protein kinase C (Prkca), which was also inferred to be downregulated in the nicotine-treated DSS mouse colon. Prkca increases Tjp1 translocation from the cell membrane to tight junctions, and inhibition of this process also indicates increased intestinal permeability in the nicotine-treated DSS model (Figure 6B). Reduced inflammation in the nicotine-treated mouse colon was supported by the inferred upregulation of transforming growth factor beta receptor (Tgfbr)2 and Thioredoxin (Txn)1, leading to decreased CD4-positive/CD8-positive T-cell activation and an inferred reduction in reactive oxygen species, respectively (Figure 6B). Moreover, inferred downregulation of the Il12 complex and Jak suggested that nicotine reduced inflammation in the colon of the DSS mouse model. In contrast, the inferred downregulation of Tlr7 leading to decreased interferon (Ifn)1b suggested that nicotine attenuated the anti-inflammatory mechanism in the colon of DSS-treated mice (Figure 6B).

Traf3ip2 (Traf3 interacting protein 2) was inferred to be downregulated, suggesting increased cell proliferation and wound healing (Figure 6B). Enhanced wound healing would also be supported by inferred upregulation of Ctnnb1 via Myc and CCnd1 with a positive input to cell proliferation. The inferred regulation of several nodes also suggested impaired would healing in the nicotine-treated mouse colitis model. MAPKp38 and Tgfb1, as well as AKT signaling, were inferred to be downregulated, signifying decreased epithelial cell migration and proliferation, respectively. The increased apoptosis suggested by the inferred upregulation of Casp8 would also have detrimental effects on wound healing in the mouse colon (Figure 6B) [37,38]. 

## 3. Discussion

Naturally occurring alkaloids in tobacco have been suggested to be potentially effective in treating several inflammatory conditions [15,26,27,39,40], but their mechanism of action and relevance to human diseases remain unclear. To clarify the impact of tobacco alkaloids on intestinal inflammation and facilitate the bench-to-bedside transition of any relevant findings, we combined a robust and well-accepted mouse UC model [41] with a highly predictive mouse model possessing a human immune system [42,43,44]. Indeed, lymphopenic NOD/Shi-scid/IL-2rγ^−^/^−^ mice transplanted with human cord-blood-derived CD34+ hematopoietic stem cells develop a naïve and functional human immune system equipped with T and B cells, NK cells, dendritic cells, monocytes, and macrophages. Finally, our study design also considered the relapsing and remitting characteristics of human CD and UC with the addition of a 5-day recovery period following the 7-day DSS treatment period (referred to as the recovery phase). This is important to evaluate the alkaloids’ effects on both the pro-inflammatory (acute) and anti-inflammatory/pro-wound healing (recovery) phases of the disease [45,46].

In this setting, our results clearly show that oral administration of nicotine and partially anatabine globally reduced the clinical manifestations of DSS-induced colitis accompanied by body weight loss, diarrhea, rectal bleeding, and colon shortening. Furthermore, we were able to link observed changes in the clinical outcomes to direct effects at the tissue level. The histopathological assessments confirmed the role of nicotine in reducing the inflammation, tissue damage, and mononuclear infiltration scores [22,23,47] during the acute phase. Finally, IHC analysis and immune cell infiltration quantification showed that both nicotine and anatabine were able to suppress increased leukocyte recruitment in the inflamed colon, which is the underlying pathophysiology of DSS-induced colitis. This effect has been described previously and linked to both nicotine-dependent downregulation of MAdCAM-1 expression and subsequent leukocyte recruitment [23] and nAChRα7 modulation [9,48,49].

The node inference and network analysis pinpointed mechanisms known to be regulated by nicotine. In line with our findings, several studies have shown that the suppressive effects of nicotine on the production of inflammatory mediators such as Tnf and Il1b occur via inhibition of the NF-ĸB pathway [47,50,51,52]. Another mechanism of Ilb activation includes the activation of Casp1, which was also inferred to be downregulated in our analysis, in line with nAchRα7 signaling inhibiting Casp1 activation [53,54]. Il1b is also positively regulated by ADRB, which was accordingly inferred to be downregulated in our analysis. This is in contrast to the findings that nicotine enhanced human colon cancer xenograft growth in mice via β-adrenoceptor stimulation [55]. However, in the context of renovascular control, nicotine was suggested to negatively regulate ADRB-mediated vasodilation [56].

The inferred downregulation of Tlr2 (upstream of the NF-ĸB complex) could result from nAchR activation, which has been shown to attenuate Tlr signaling. Topically administered nicotine was shown to decrease Tlr2 production in diabetic wounds infected with bacterial agents and reduce the inflammatory response during skin wound healing [57,58].

Our analysis also predicted nicotine-mediated downregulation of Tlr7 in DSS-treated mouse colon. According to the network model, Tlr7 downregulation leads to downregulation of Ifnb1, which inhibits anti-inflammatory mechanisms. Although this is initially counterintuitive, the evidence shows opposite effects elicited by TLR7 activation/inhibition in the context of inflammation. Although TLR7 activation has been shown to protect mice from DSS colitis by mediating the action of enteric viruses [59,60], its expression in colonic mucosa is increased following antibiotic-induced dysbiosis in mice [61]. Thus, TLR signaling inhibition is now considered a promising therapy for inflammatory-mediated diseases [62] such as systemic lupus erythematosus [63], psoriasis [64], autoimmune uveitis [65], and pancreatic cancer [66].

MAPKp38 is connected to epithelial cell migration in our IBD network models, and its inferred downregulation would indicate impaired wound healing. However, some studies have suggested that MAPKp38 is activated in the inflamed intestinal mucosa [67], and its inhibition suppresses IBD [68]. The involvement of MAPKs in the favorable effects of nicotine on DSS-induced colitis was also highlighted by the inferred downregulation of Map3k5 that would, according to the IBD model, decrease translocation of Ocln and Tjp1 from tight junctions to the cell membrane. This is in line with previous findings showing that the favorable effects of nicotine on burn-induced intestinal permeability were mediated by decreased expression and mislocalization of Ocln and Tjp1 by nicotine [69]. Moreover, vagal nerve stimulation attenuated the endotoxemia-induced loss of Ocln and Tjp1, which was blocked by α-bungarotoxin, indicating the involvement of nAchRα7 [70]. Based on our analysis, the observed nicotine effects on tight junctions could be mediated by Map3k5.

In the IBD network model, vitamin D signaling downregulates the NF-κB component, Rela [71], and its inferred upregulation by nicotine is in line with the inferred downregulation of the NF-κB complex by nicotine in the DSS-treated mouse colon. Vitamin D deficiency has been linked to IBD in humans [72], and both mouse and cell models have shown that vitamin D protects the intestinal epithelial barrier [71,73]. The mechanism by which nicotine regulates vitamin D signaling is not known.

The study has its limitations. Due to the lack of an exposure arm without DSS treatment in the study design, it was not possible to investigate how tobacco alkaloids may impact the healthy colon in the humanized mouse model. Moreover, analysis of the human-cell-derived transcriptome was not possible due to the small sample sizes extracted from the colon of the alkaloid-treated DSS mice. Even though the changes in immune cell infiltration to the colon at day 7 were marginal in the tobacco-alkaloid- and DSS-treated groups compared to mice treated with DSS only, the immune cells likely signal to the colon epithelium. Hence, the analysis of the immune cell transcriptome in the humanized mouse model could further explain the inferred molecular changes observed in the colon transcriptomic analysis and increase understanding of how tobacco alkaloids protect the mouse colon. Nevertheless, our results can be used to generate limited hypotheses on the mode of action of nicotine in experimental colitis, which should be confirmed with additional investigations.

In summary, we demonstrated that administration of nicotine in drinking water reduced colitis clinical symptoms and histologic features in a DSS-mouse model with a fully humanized immune system. The underlying molecular mechanisms were linked to known effects of nAchRα7 signaling, and the node inference also suggested novel mechanisms of nicotine action that could be further explored.

## 4. Materials and Methods

### 4.1. Animal Experimentations

All animal procedures described in this study were reviewed and approved by the local ethic committee (CELEAG–TCS agreement number A7418324. 02NOV2020: APAFIS#26859-2020092416353236 v4; 23NOV2017: APAFIS#9260-2017031514075189 v2). The experiment was carried out with NOD/Shi-scid/IL-2Rγnull immunodeficient mouse strain (NCG from Charles River Laboratories, Wilmington, MA, USA). Briefly, 4-week-old immunodeficient NCG mice were engrafted with cord-blood-derived human CD34+ hematopoietic stem and progenitor cells (French Blood Institute, Paris, France) 2 days after chemical myeloablative treatment. Fourteen weeks after cell injection, engraftment level was monitored with the analysis of human CD45+ cells among total blood leukocytes by flow cytometry (Attune, Life Technologies/Thermo Fisher Scientific, Waltham, MA, USA). After engraftment confirmation, mice were assigned to each treatment group after manual randomized based on their humanization rate, their weight, and the cord blood donor ID.

### 4.2. DSS-Induced Acute Colitis

Acute colitis was induced by adding DSS (40 kDa; Sigma-Aldrich, St. Louis, MO, USA; Cat. No. 42867) in drinking water over 7 days, from day 1 to day 7. DSS powder was aliquoted on the first day of the experiment. DSS solution was freshly prepared by dissolving the aliquoted powder in MilliQ water (final concentration of 3%), and it was renewed every 2 days. For the washout period, the DSS solution was removed and replaced by MilliQ water from day 8 to day 12. The progression of the disease severity was monitored with clinical scoring. Mice were weighed daily during the acute colitis cycle and scored for their stool consistency and rectal bleeding by visually monitoring stool. Using these parameters, a global clinical score was calculated (sum of the body weight score, diarrhea score, and bleeding score. Additional data visualizations and detailed protocols are available on the INTERVALS platform at https://doi.org/10.26126/intervals.7p6n5w.1.

### 4.3. Treatments

Treatments started 2 weeks before colitis induction by DSS and were maintained until sacrifice. Nicotine solution (Sigma-Aldrich; N3876) and anatabine (custom synthesized by WuXi Apptech, Shanghai, China. Research grade (scale synthesis of API) with a purity of 99%) were added to the drinking water to reach a value of 20 mg/kg/day.

### 4.4. Flow Cytometry

Flow cytometry analysis was performed on an Attune N × T Flow Cytometer (Life Technologies/Thermo Fisher Scientific). Briefly, 100 µL of blood was stained with surface antibodies (using anti-hCD45, hCD14, hCD16, hCD15, hCD86, hCD19, hCD163, and hCD11b fluorophore-linked antibodies from Miltenyi, Bergish Gladbach, Germany) and viability dye. Red blood cells were lysed with RBC lysis buffer (eBioScience, San Diego, CA, USA). Samples were then permeabilized with Fixation/Permeabilization Buffer (Thermo Fisher Scientific) for the intracellular staining. Washing steps preceded cytometer analysis. The numbers of samples were n = 10 at D7 for all groups, n = 7 for the water and anatabine groups at D12, and n = 10 for the nicotine group at D12.

### 4.5. Nucleic Acid Extraction

Distal colon tissues were collected in Magnalyser tubes (Roche, Basel, Switzerland) and ground in 700 µL of Qiazol lysis buffer (Qiagen, Hilden, Germany) with the FastPrep-24 5G (MPBio, Irvine, CA, USA). Total RNAs (including microRNAs) were purified using miRNeasy Mini Kit (Catalog number 217004, Qiagen) according to the manufacturer’s instructions.

RNA concentration and purity were determined using a UV spectrophotometer (NanoDrop ND1000, Thermo Fisher Scientific) by measuring the absorbance at 230, 260, and 280 nm. The integrity of the RNA was further checked with the Agilent 2100 Bioanalyzer, and RNAs with RIN values > 7 were used in the downstream RNAseq workflow.

### 4.6. RNA Sequencing

The RNA was normalized to 200 ng for each sample, and the library was prepared with the Tecan Universal Plus mRNA-Seq Kit (Tecan, Männedorf, Switzerland). Briefly, this kit generates stranded RNA-Seq libraries derived from poly(A)-selected RNA. The PolyA RNA was reverse-transcribed and transformed into a double-stranded cDNA. Unique dual-indexed adapters were ligated, and libraries were then amplified with 10 polymerase chain reaction cycles and purified. The quality of the libraries was also checked on a Fragment Analyzer 96 instrument (Agilent Technologies, Santa Clara, CA, USA). The purified libraries were then quantified with NuQuant and normalized to 10 nM. Eight sequencing pools were clustered on a c-Bot instrument and sequenced on the Illumina HiSeq 4000 Instrument (Illumina, San Diego, CA, USA).

### 4.7. Histology and Immunohistochemistry

Colons were dissected, measured, and fixed in 4% paraformaldehyde for 24 h before storage in 70% ethanol. Samples were dehydrated before paraffin embedding and trimming using a Leica TP1020 Tissue Processor (Leica, Wetzlar, Germany). Each colon sample was embedded in one paraffin block to perform longitudinal sections. Sections of 4 μm per block were deposited on Superfrost + slides before HE staining. Two sections of the same block were used to prevent artifacts from manual coloring. Analysis was performed on mice colon tissues prepared with the Swiss-roll technique. All slides were digitalized using the scanner Aperio Versa (Leica) with the ×20 objective in brightfield conditions. Inflammation/fibrosis, tissue damage, and mononuclear cell infiltration were then visually assessed in proximal, middle, and distal regions and scored (from 0 to 4, based on Table 1), and a percentage of surface was measured using ImageJ software (National Institutes of Health, Bethesda, MD, USA). Finally, colons were analyzed with IHC to detect T cells (CD4+, CD8+), B cells (CD19+), macrophages (CD68+), neutrophils (CD66b+), and proliferating cells (Ki-67+) (list and dilutions of antibodies provided in Table 2). Primary antibodies were incubated for 30 min and secondary antibodies were incubated for 10 min before chromo detection (DAB) for 5 min. IHC was performed in one section per mouse. On each section, three areas were checked (proximal, middle, and distal), and three images per area were analyzed. The number of positive cells per image was counted using the ImageJ Fiji package; this number was normalized to the total number of cells (quantified with the number of nuclei) and shown as the percentage of positive cells.

### 4.8. Data Analysis

All parameters were analyzed using GraphPad Prism software (version 7; GraphPad Inc., San Diego, CA, USA). One-way ANOVA with Dunnett’s multiple comparison test was used, and the results are shown in Figure 2I,J, Figure 3C, Figure 4, and Figures S2 and S3. Two-way repeated measures ANOVA with Geisser–Greenhouse and Dunnett’s multiple comparison tests was used for Figure 2A–H. Two-way ANOVA with Sidak’s multiple comparison test was used for Figure 5. All the treatment groups were compared to the vehicle group.

### 4.9. Transcriptomic Data Analysis

The raw sequencing data were first cleaned to remove low-quality and adapter sequences. Downstream analysis revealed that mouse RNA was much more abundant than human RNA in the collected samples (at least 100-fold, see Supplementary Figure S4A). A two-fold mapping strategy to map the joint human and mouse reference transcriptomes was applied (HGNC and MGI gene symbols, respectively). In the first run, reads mapping equally well to human and mouse orthologous genes were discarded, whereas in the second run, these “shared” reads were counted in both the human and mouse orthologous genes. For a human gene with a mouse ortholog, the two obtained count values $n_{Hs,1}$ and $n_{Hs,2}$ enabled estimation of the actual human count value $n_{Hs,\mathrm{actual}}$ by splitting the shared reads $n_{Hs,\mathrm{shared}}$ according to their estimated human fraction $\alpha_{Hs}$ (expressed in terms of the respective human and mouse “specific” counts values $n_{Hs,1}$ and $n_{Mm,1}$):

$$\left\{\begin{array}{ccc} n_{Hs,\mathrm{actual}} & = & n_{Hs,1}+\alpha_{Hs}\cdot n_{Hs,\mathrm{shared}}\\ \alpha_{Hs} & = & \frac{n_{Hs,1}}{n_{Hs,1}+n_{Mm,1}}\\ n_{Hs,\mathrm{shared}} & = & n_{Hs,2}-n_{Hs,1} \end{array}\right.$$

An analogous approach was applied for the corresponding mouse gene, even though the contamination effects by the much less abundant human ortholog gene were expected to be weaker.

Mapped transcriptomic data were processed in the R software environment for statistical computing [74]. The human and mouse orthologous gene relationships were obtained from the NCBI website (https://www.ncbi.nlm.nih.gov/homologene, accessed on 10 September 2019). The validity of the mapping strategy was assessed by running an over-representation analysis of the top 1000 genes contained in each tail of the distribution of the log10 ratio between the mean read counts in human and mouse for orthologous genes. The tissue-specific gene set collections “Human_Gene_Atlas” and “Mouse_Gene_Atlas” were taken from the EnrichR website (https://maayanlab.cloud/Enrichr/, accessed on 10 September 2019). The gene set over-representation calculation was performed using the piano package [75]. Once the actual human and mouse read count matrices were calculated and validated, a standard edgeR-based RNAseq analysis pipeline was run to obtain the gene differential expressions.

Node enrichment analysis was used to identify molecular drivers of nicotine effects on DSS-induced colitis. The strength scoring algorithm is a threshold-free enrichment method that relies on mRNAs known to be regulated by specific nodes, as described in [34,76]. The nodes with an inferred adjusted *p*-value < 0.05 were then mapped to the IBD network model suit using Cytoscape [77]. The graphs were further edited for better readability using Adobe Illustrator (Adobe, San Jose, CA, USA) [78]. The IBD network models are available for viewing and download as reported in [37,38].

## Figures and Tables

**Table 1:** Pathology score.

Score	Inflammation Score	Inflammation Extent	Mucosal Damage Score	Mucosal Damage Extent	Density Score of Mononuclear Infiltration
0	None or residual	% surface	None	% surface	None
1	Minimal (small scattered foci of inflammation)		Focal crypt lesion, minimal erosion but not ulceration		Minimal
2	Slight (slightly larger and/or more foci)		Extension in the mucosa (large erosion and/or ulceration) involving at most half of mucosal thickness		Slight (several clusters and aggregate or minimal but diffuse)
3	Moderate (relatively marked but multifocal in mucosa)		Extensive damage affecting more than half of the mucosa (50/75%); loss of epithelium		Moderate density (numerous clusters of slight and diffuse)
4	Marked transmural		Extensive damage to the entire mucosa or almost		Marked density

**Table 2:** List of antibodies and references.

Antibodies	Dilution	Reference	Company ^a^
Anti-CD4 (EPR6855)	1/500	ab133616	Abcam
Anti-CD8 (SP16)	1/500	108R-14	Invitrogen
Recombinant anti-CD68 (EPR20545)	1/6500	ab213363	Abcam
Anti-CD19 antibody	1/300	ab134114	Abcam
Ki-67 monoclonal antibody (SP6)	1/1200	MA5-14520	Invitrogen
Anti-CD66b	1/300	ab214175	Abcam
Anti-rabbit IgG	1/2000	ab205718	Abcam

^a^ Abcam (Cambridge, UK), Invitrogen (Carlsbad, CA, USA).

## Data Availability

Datasets, additional data visualizations, and detailed protocols are available on the INTERVALS platform at https://doi.org/10.26126/intervals.7p6n5w.1.

## Supplementary Materials

The following supporting information can be downloaded at: https://www.mdpi.com/article/10.3390/ijms24076419/s1.
