# Supplementary material for: Tobacco Alkaloid Assessment in a DSS-Induced Colitis Mouse Model with a Fully Humanized Immune System
Source: Int J Mol Sci. 2023 Mar 29;24(7):6419. doi: 10.3390/ijms24076419 (PMC10095104; doi:10.3390/ijms24076419)
Supplement: Supplementary file 1 [file ijms-24-06419-s001.zip › Lo Sasso et al._Supplementary Material_Final.docx]

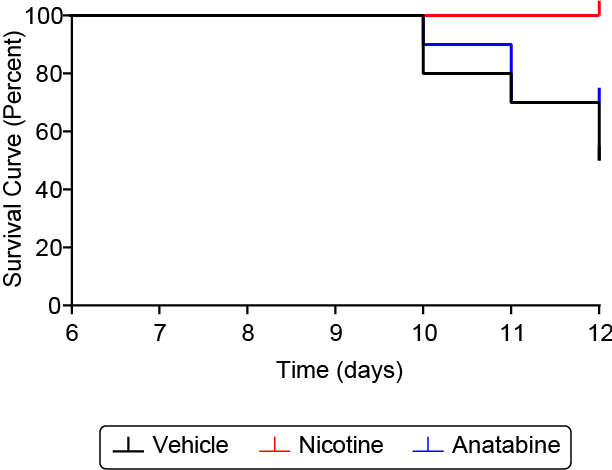


## Supplemental Figure 1: Nicotine protects against DSS-induced mortality.

Kaplan-Meier survival curves, n = 10/group at the beginning of the experiment. Nicotine and anatabine were given in the drinking water at 20 mg/kg/day.


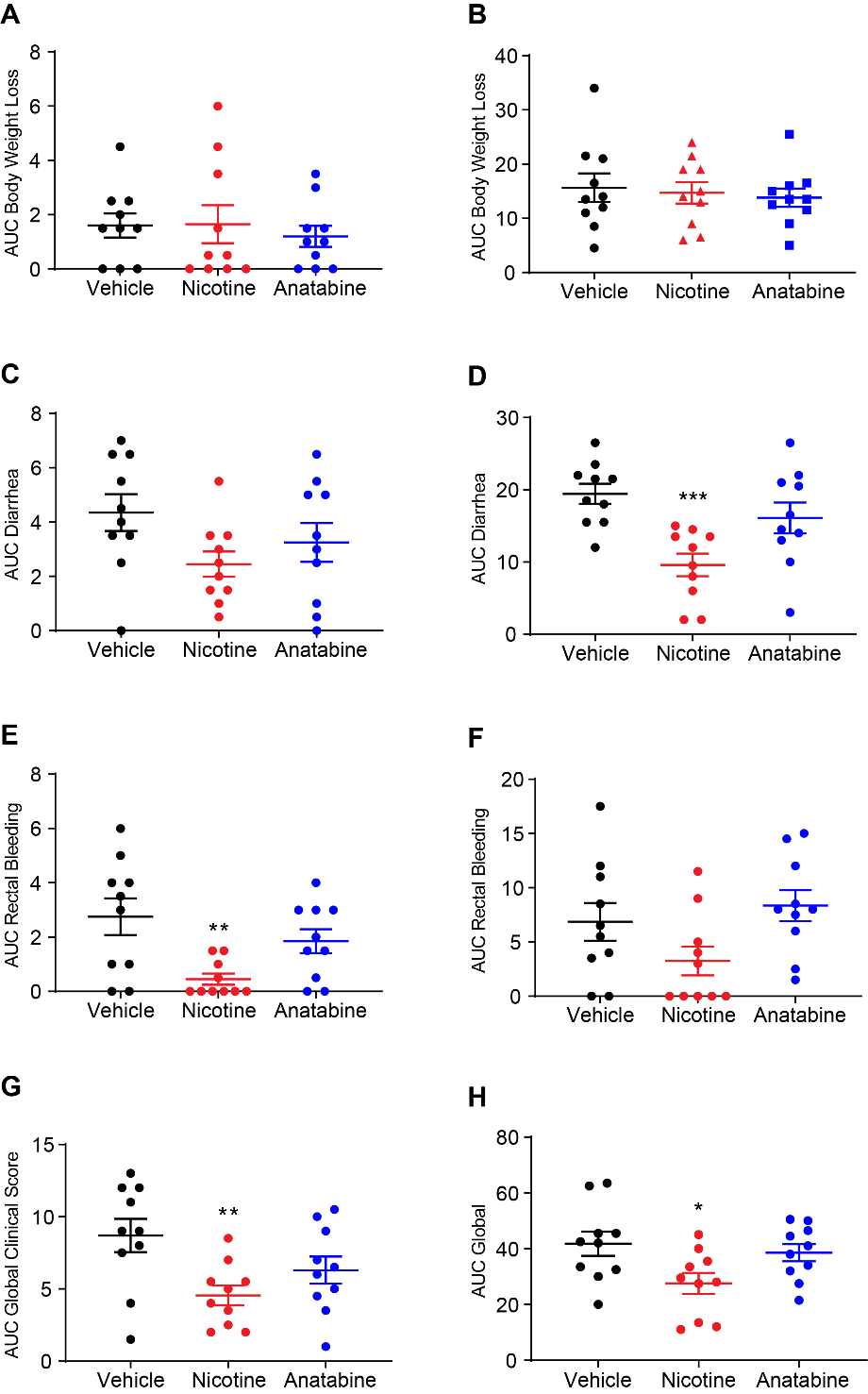


## Supplemental Figure 2: Effect of tobacco alkaloids on clinical parameters of DSS-induced colitis.

Areas under the curve (AUCs) from D1 to D7 (acute phase, A, C, E, G) and D1 to D12 (recovery phase, B, D, F, H) for body weight loss (A, B), diarrhea (C, D), rectal bleeding (E, F) and global clinical score (G, H). Results are expressed as mean ± SEM, n = 10/group at the beginning of the experiment. *p ≤ 0.05, **p ≤ 0.01, ***p ≤ 0.001. D7: N= 10 for all groups. D12: N=7-9 for Vehicle and Anatabine; N=10 for nicotine.


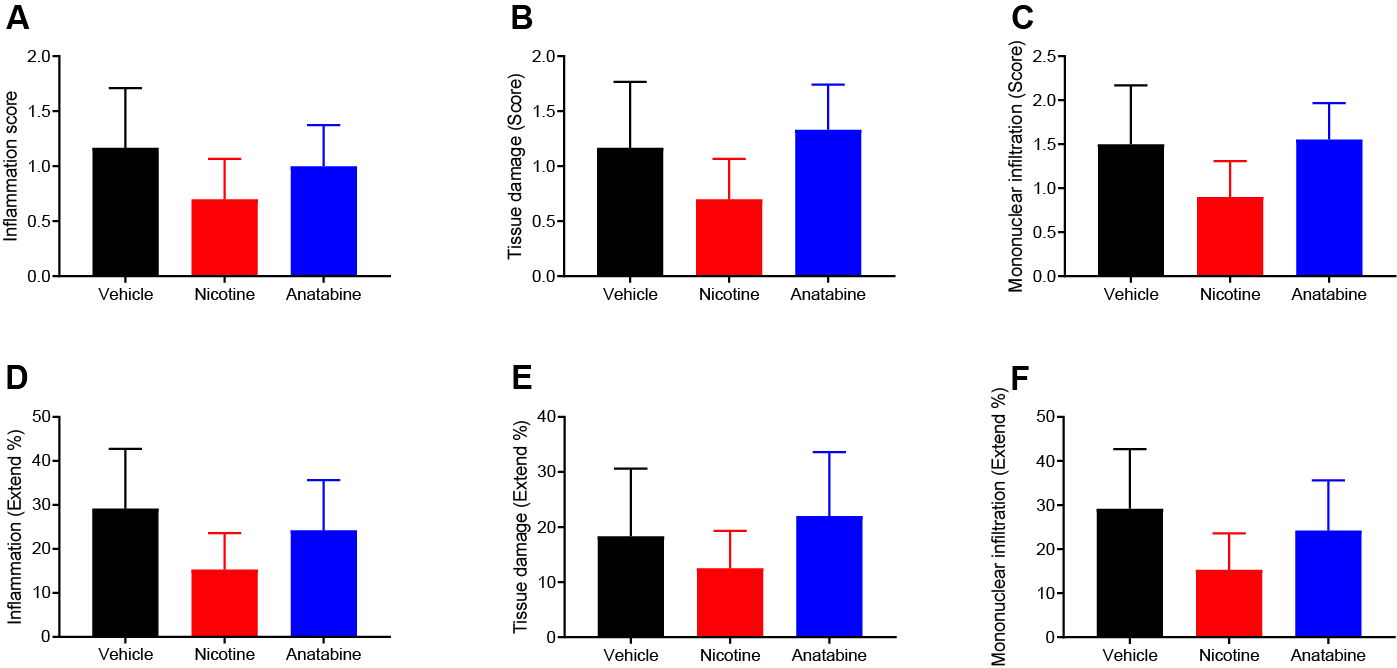


## Supplemental Figure 3: Nicotine reduces histological features of UC at D12.

Inflammation (A, D), tissue damage (B, E), and mononuclear cell infiltration (C, F) score (A, B, C) and extent (D, E, F) in the distal part of the colon. Mice were sacrificed at the end of D12 for histology assessment. Mean and SEM are shown, n = 7-10.


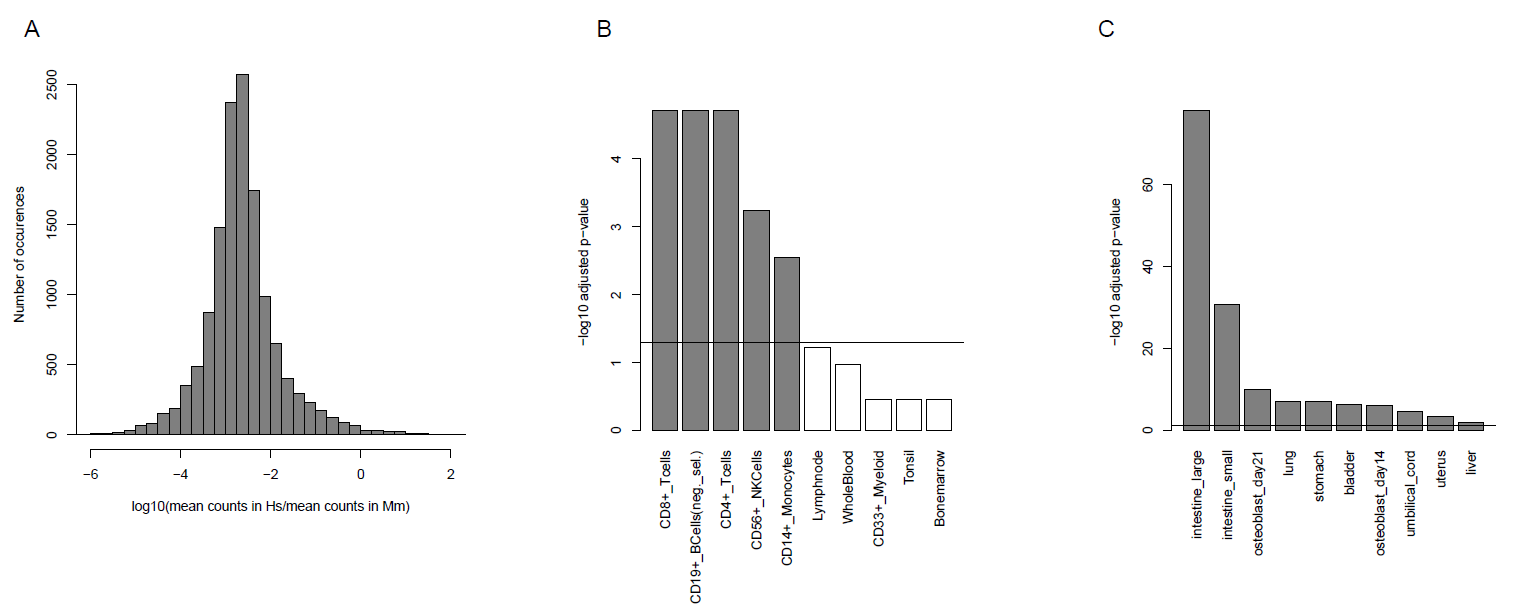


## Supplemental Figure 4: RNA sequencing data preprocessing.

(A) Histogram of log10 ratios between the mean read counts in human (Hs) and mouse (Mm) for all orthologous genes. By fitting a smooth curve to the histogram, the most likely value was found at about -2.71, which corresponded to a ~1:500 count ratio between human and mouse genes. (B) Bar plot of the top-10 results (adjusted log10 p-values) of the over-representation calculation for the “Human_Gene_Atlas” collection. The 1000 “human-specific” genes located in the left-hand tail of Panel A) were used for the over-representation calculation. The standard threshold for statistical significance was chosen as 0.05 and is indicated by the horizontal line. (C) Bar plot of the top-10 results (adjusted log10 p-values) of the over-representation calculation for the “Mouse_Gene_Atlas” collection. The 1000 “mouse-specific” genes located in the right-hand tail of Panel A) were used for the over-representation calculation. The standard threshold for statistical significance was chosen as 0.05 and is indicated by the horizontal line.
