# Supplementary figures and images for: Tobacco Alkaloid Assessment in a DSS-Induced Colitis Mouse Model with a Fully Humanized Immune System
Source: Int J Mol Sci. 2023 Mar 29;24(7):6419. doi: 10.3390/ijms24076419 (PMC10095104; doi:10.3390/ijms24076419)

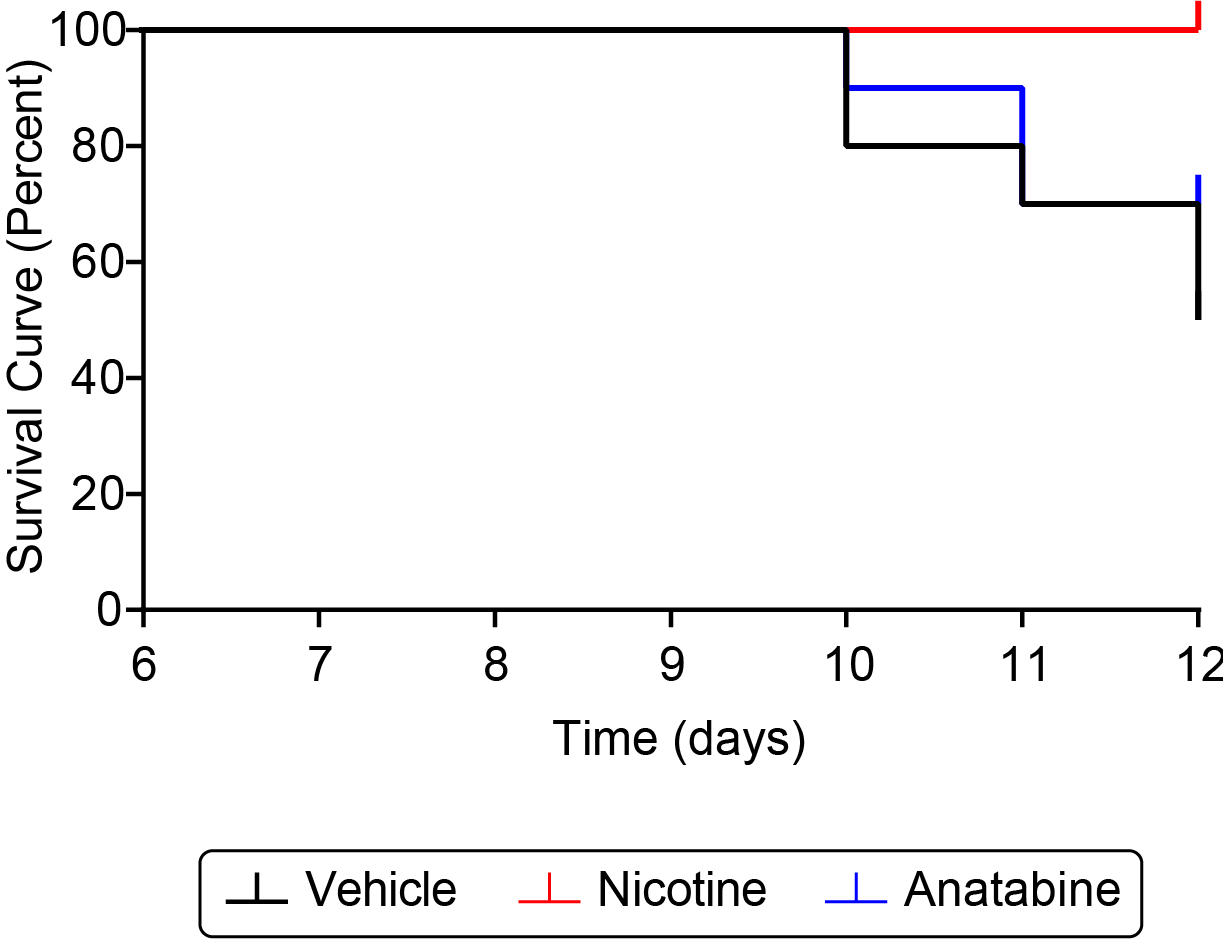

Supplement: Supplementary file 1 [file ijms-24-06419-s001.zip › Supplementary Figure 1.tif]

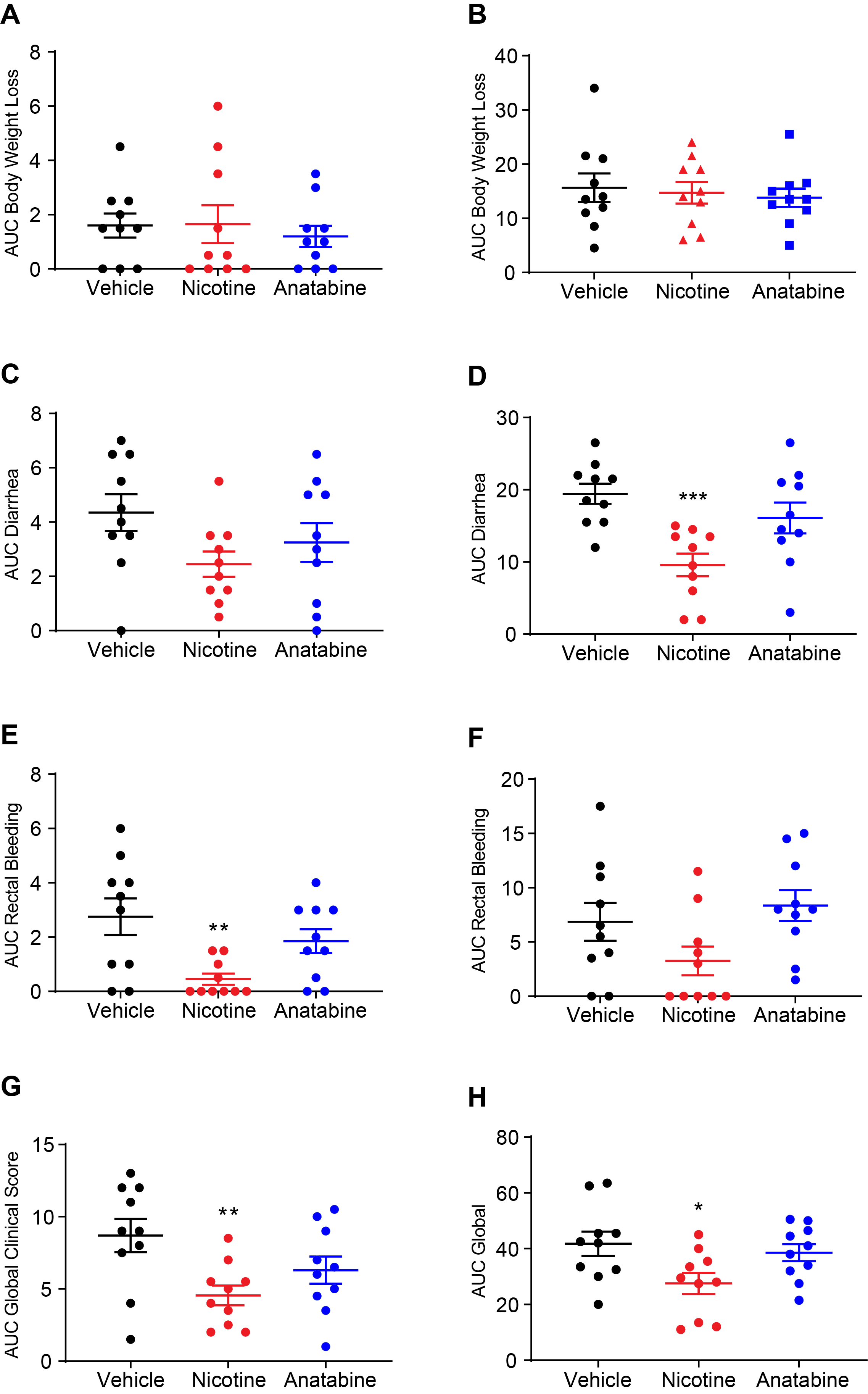

Supplement: Supplementary file 1 [file ijms-24-06419-s001.zip › Supplementary Figure 2.tif]

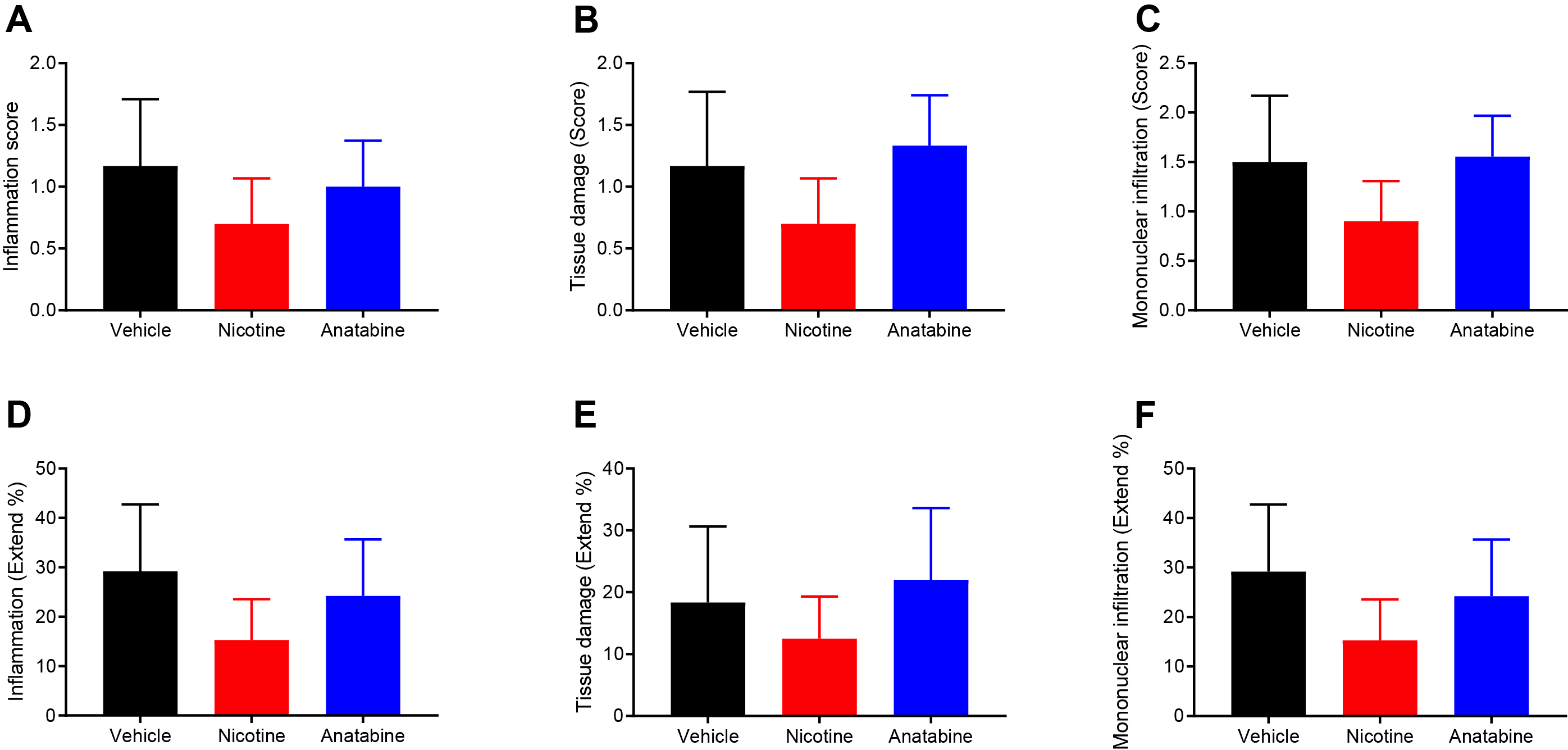

Supplement: Supplementary file 1 [file ijms-24-06419-s001.zip › Supplementary Figure 3.tif]

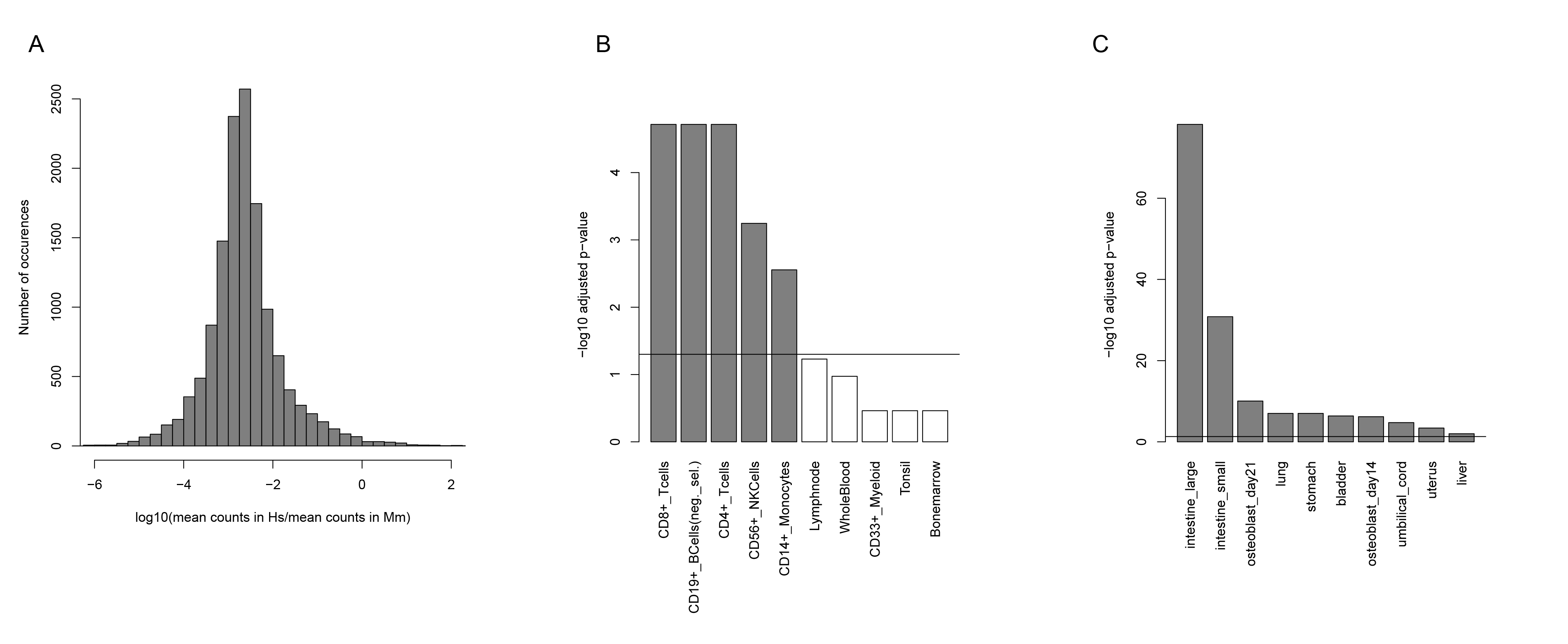

Supplement: Supplementary file 1 [file ijms-24-06419-s001.zip › Supplementary Figure 4.tif]
